# Endovascular Materials and Their Behavior in Peripheral Vascular Surgery

**DOI:** 10.3389/fsurg.2022.900364

**Published:** 2022-05-04

**Authors:** Daniela Mazzaccaro, Matteo Giannetta, Paolo Righini, Alfredo Modafferi, Giovanni Malacrida, Giovanni Nano

**Affiliations:** ^1^ Operative Unit of Vascular Surgery, IRCCS Policlinico San Donato, San Donato Milanese, Milan, Italy; ^2^ Department of Biomedical Sciences for Health, Università degli Studi di Milano, Milan, Italy

**Keywords:** endovascular, peripheral arterial disease, materials, femoro-popliteal, iliac

## Abstract

Endovascular techniques have progressively become the first option for the treatment of stenosis and occlusions of both aorto-iliac and femoro-popliteal district. The development of new technologies and new materials has broadened the applicability of the endovascular techniques, allowing the treatment of each lesion with the most suitable material. A knowledge of the behavior of endovascular materials when treating peripheral arterial disease (PAD) is, therefore, crucial for optimization of the results. Here, we aim to review the most important technical features of the actually available endovascular materials for treating PAD.

## Introduction

Atherosclerotic peripheral arterial disease (PAD) is associated with an increased risk of limb loss (1). Therefore, it needs prompt risk factor management and optimal pharmacological treatment. Appropriate revascularization may also be required, especially in the case of progressive clinical deterioration despite optimal medical therapy or in the presence of severe limiting claudication with reduced quality of life, rest pain, and/or critical ischemia (2).

PAD may affect every segment, right from the infrarenal aorta and iliac arteries to the femoral, popliteal, and tibial arteries, extending even to the smaller foot arteries. A tailored approach is then required based on individual clinical risk and on the site of the arterial disease.

Endovascular techniques have progressively gained wide acceptance as the first option in the revascularization of short stenosis/short occlusions of both aorto-iliac and femoro-popliteal district (3), given their low invasiveness and better outcomes when compared with open surgery.

Technical improvement of the devices, along with the development of new technologies, have increased the applicability of the endovascular techniques, allowing the treatment of each lesion with the most suitable material.

A knowledge of the behavior of endovascular materials when treating PAD is, therefore, crucial for optimization of the results.

Here, we aim to review the most important technical features of the actually available endovascular materials for treating PAD.

## Type of Endovascular Devices

A wide variety of guidewires, catheters, crossing devices, balloons, stents, and other devices are available for the endovascular treatment of PAD, but there are no clear guidelines for their application.

When planning the best endovascular strategy, the operator should consider the type and anatomic location of the target lesion, within the context of the whole vessel. Some scoring systems have also been elaborated to help in the preoperative technical stratification of femoropopliteal and infrapopliteal artery disease treatment, such as the Bollinger score, the TASC II grade, the run-off score, the calcium score, and the lesion length category (4). These scores describe the anatomical features of the index lesion and are helpful tools in predicting durability, outcomes, and response to endovascular interventions. Therefore, they should be taken into account when choosing the proper treatment.

### Guidewires

Guidewires are the mainstay of endovascular treatment, since they are used basically in every procedure from the beginning to the end, to reach and cross the target lesion, and as a support for therapeutic devices such as balloons or stents.

Each guidewire has its own engineering features in terms of maneuverability, flexibility, visibility, traceability, smoothness, and support. The choice of the correct guidewire can be crucial for obtaining the optimal result, especially in the case of chronic total occlusions (CTOs).

Different guidewires are available in terms of core diameters, lengths, core material, tip design, covers, and coating. All these features may impact the strength, the flexibility, and the trackability of the wire.

The core diameter can range from 0.014 to 0.038 in., but in peripheral procedures, the most widely utilized sizes are 0.014, 0.018, and 0.035 in. The larger the diameter, the grater the rail support is. Conversely, larger diameter guidewires have reduced flexibility and trackability through the vessel (5). Usually, 0.035 in. guidewires are used for delivering sheaths and diagnostic catheters, while 0.018 in. are used for crossing more proximal lesions, and 0.014 in. are used for below knee interventions and CTOs.

The length of a guidewire can reach up to 300 cm. Usually, shorter guidewires are easier to maneuver and have a greater pushability to cross the lesion. Nevertheless, device compatibility and a knowledge of the road from the access vessel to the target lesion should be considered when choosing the proper guidewire diameter and length.

The most used guidewires are Nickel Titanium (Nitinol), which combine the columnar support of a stainless-steel guidewire with flexibility and good trackability. The tip, straight or “J”-shaped, can be made either of the same core material, giving a greater push force, or may be more delicate and soft, with a lesser probability to inadvertently damage distal vessels.

Finally, guidewires can be covered and coated by sleeves of polymer or plastic to increase lubricity, therefore, providing an enhanced lesion crossing and smooth tracking, especially in tortuous vessels. The most used are hydrophilic coatings to create a slippery surface that facilitates navigability through the vessels.

### Catheters

Diagnostic and guiding catheters enable direct access to the treatment site when guidewires fail.

Depending on the case, they have specific preformed shaped tips at their distal ends that can help in intra-arterial navigation, correct orientation of the tip of the guidewire toward the lesion site, crossing of complex lesions, and opacification of the artery using a contrast agent.

The diameter unit of the catheters is measured in French (1 French = 0.3 cm). The length of the guidewire should always be greater than that of the catheter used.

### Crossing Devices

CTOs are complex lesions that may deserve specific equipment with its own behavior.

Therefore, crossing devices are available for the endovascular antegrade and retrograde recanalization of peripheral CTOs, either under fluoroscopy or under intravascular sound guidance.

The aim of a crossing device is to perform microdissection and disruption of the atherosclerotic plaque while advancing the distal tip, which can have specific features (such as jaws or edges or may be connected to a generation of vibration energy) that can help penetrate through the CTO (6). While advancing through the CTO, a microguide catheter is sometimes needed to provide support to the distal end and guidewire exchange.

After having crossed the CTO, a re-entry device may be needed to reach the true distal lumen from the subintimal plane beyond the CTO. These devices usually have a hollow curved needle or a microcatheter lancet at their tip. After re-entering the true lumen, guidewire placement is needed for completion of the procedure using balloons and stent delivery.

### Balloons

Balloon angioplasty can be either coated or noncoated by drugs that aim specifically at reducing the risk of restenosis caused by neointimal hyperplasia (4).

Typical features of an angioplasty balloon that can affect one’s behavior during endovascular procedures include geometry, cutting ability, and the fabric material.

The geometry and the fabric material may impact the crossing profile. Balloons with a low crossing profile are for ease of entering and crossing challenging lesions and can be a useful tool to prepare the road for greater balloons. Furthermore, the geometry and the material are crucial for length and radial compliance of the balloon, which, in turn, may affect the rate of inflation and deflation.

The pressure exerted with the inflation allows the balloon to reach its nominal diameter or even a few tens of a millimeter more. However, inflation pressure cannot exceed the rated burst pressure. Usually, short balloons may need more pressure to reach their nominal diameter, but they can have a higher radial force for short lesions. Conversely, long stenotic regions may need longer balloons that may require a longer time of application.

In highly fibrotic lesions, such as postattinic stenosis or restenosis, balloons with small cutting edges (cutting balloon) can be employed to fracture the plaque.

### Stents

According to the method of deployment, stents can be premounted on a balloon (balloon-expandable) or may have their own delivery system with a self-expandable opening.

Usually, balloon-expandable stents have a greater radial force, a greater radiopacity, and a more precise delivery when compared with self-expandable stents (7). Conversely, they have lower flexibility and trackability than self-expandable stents. Therefore, balloon-expandable stents are indicated for short, calcified stenosis, while self-expandable stents perform better in long lesions and tortuous arteries.

Furthermore, each stent may have its radial force or resistance to elongation, torsion, and crushing according to the struts material and shape. The struts may have bridges that connect to each other, leading to a design with “closed-cells.” Otherwise, the struts may have large uncovered gaps in a “open-cells” configuration. The number and frequency of bridges of the struts confer less flexibility and trackability to the stent.

When using balloon-expandable devices, a small risk of decrimping the stent from the balloon while crossing an occlusion or tightly calcified stenosis should be considered. Therefore, predilation with a smaller balloon is suggested. During expansion, the stent will take on the diameter of the balloon on which it is mounted. However, if needed, balloon-expandable stents may be dilated to a greater diameter by using a larger diameter balloon but that is shorter than the stent, in order to avoid dissection of the artery at the proximal and distal ends of the stent.

Differently, self-expanding stents cannot exceed their reference diameter. Therefore, when in doubt choosing between two options, it is preferable to opt for larger diameter stents. These stents open only slightly after delivery due to their low radial force, and, therefore, postdilation is needed.

Of note, self-expanding stents may act on the vessel wall in terms of chronic outward force, which is the radial force that the stent exerts at expansion, and is proportional to the amount of oversizing with respect to the vessel diameter. This amount of oversizing can induce wall shear stress, which can be responsible for in-stent restenosis; therefore, excessive oversizing should be avoided when choosing the proper stent diameter.

While the indication of stent placement in the aorto-iliac district is well recognized (7), conflicting data exist about the placement of a stent in the superficial femoral artery (SFA) and popliteal districts, since stent fatigue may occur, especially in Nitinol stents, leading to stent failure and vessel reocclusion (8).

The mechanical behavior of stents in the SFA-popliteal artery, which has been studied using the spring model and 3D finite element modeling, depends not only on the technical features of the stent, but also on the torsion and elongation of the vessel itself, to which the stent is subjected during the normal deambulation process. The type of the lesion is also an important determinant. Furthermore, overlapping regions and overexpansion of the stent beyond its nominal diameter may increase stent fatigue with the consequent risk of fracture and failure (8).

Balloon-expandable and self-expandable stents may be covered by poly-tetrafluoroethylene (covered stents) or bonded with heparin or drugs that are slowly released over time to the endothelium (drug-eluting stents, discussed below), with the aim of improving long-term vessel patency.

## Recent Technologies

### Atherectomy Devices

Atherectomy devices represent a new way of catheter-based intervention for the treatment of PAD, based on the disruption of the plaque with different physical methods.

All of these devices have in common the presence of a catheter that is inserted inside the vessel lumen, and at the tip, there are specific devices for plaque debulking. Orbital atherectomy devices have a tip with a diamond-coated crown that rotates 360° in eccentric fashion, while rotational atherectomy uses rotating cutting blades at high speed. Directional atherectomy devices also have conical rotating blades at the tip, which has in adjunction a nose cone in which the removed debris of the plaque are captured and stored. Finally, laser atherectomy devices use the physical properties of the laser wave to debulk the lesions (5).

Atherectomy is usually performed as an adjunctive therapy with balloon angioplasty and stenting.

### Lithotripsy

Intravascular lithotripsy is a novel technique for lesion preparation in calcified vessels. Lithotripsy is composed of a catheter that is connected to a generator and is enclosed in a semicompliant balloon. Electric sparks produced by the emitters create vapor bubbles in the surrounding fluid medium, resulting in acoustic pressure waves, which, in turn, create micro–macro fractures on endothelial calcium, without affecting soft tissue (5).

### Drug-Coated Devices

Balloons and stents may be covered by cytotoxic drugs that aim to reduce the inflammatory response of injured endothelial cells and subsequent neointimal hyperplasia. Paclitaxel is the most used of these drugs and arrests the cells in the M phase of the mitotic cycle. Sirolimus has also been recently approved for use in PAD (9).

What makes the difference between the different kinds of available drug-coated devices is the carrier system, which ideally should both prevent the loosing of the drug while crossing the sheath and navigating the vessels and confer rapid transfer from the balloon surface to the arterial wall when the target lesion has been reached.

### Focal Self-Expanding Nitinol Stents

Recently, a multiple stent system for spot stenting has been introduced for the treatment of femoro-popliteal lesions, with promising early results (10).

This device has six short (13 mm in length) self-expanding nitinol stents on a single 6F delivery system that is compatible with a 0.035 in. guidewire, and each of them can be individually implanted. The peculiarity of having multiple short stents instead of longer ones has the advantage of reducing the mechanical strain to which the stents can be subjected by bending and stretching, especially in the distal SFA or the popliteal artery.

Sigl et al. (10) described the first-in-human experience in 20 patients affected by claudication or critical limb ischemia, who underwent femoropopliteal revascularization using this device. They reported promising results with no device-related complications, no major adverse events, and a 100% patency at 6 months’ follow-up.

### Chocolate™ PTA Balloons

The Chocolate™ PTA balloon has a unique design. It is a semicompliant balloon that is encased in a nitinol-constraining structure (cage). When the balloon inflates, the cage expands simultaneously, causing the segmentation of the balloon in a series of pillows and grooves with controlled dilatation on the vessel wall that allows for 1:1 vessel sizing.

The over-the-wire platform is compatible with 0.014 and 0.018 in. guidewires and is available in diameters ranging from 2.5 to 6 mm, therefore, allowing treatment both below and above the knee districts, with promising early results. Sirignano et al. (11) treated 81 claudicant patients who had femoro-popliteal lesions and reported a primary patency of 98.8% at a mean follow-up of 12.3 ± 5.6 months.

Furthermore, data obtained from the Chocolate BAR multicenter postmarket registry (12) showed a 97.2% rate of freedom from major amputation at 12-months’ follow-up in 262 patients.

## Conclusion

Newer technologies are rapidly becoming available for the endovascular treatment of PAD. Endovascular operators should have a proper knowledge of the behavior of these materials in order to optimize the results.
